# Ru-catalyzed dehydrogenative coupling of carboxylic acids and silanes - a new method for the preparation of silyl ester

**DOI:** 10.3762/bjoc.4.27

**Published:** 2008-07-30

**Authors:** Guo-Bin Liu, Hong-Yun Zhao

**Affiliations:** 1 Department of Chemistry, Fudan University, 220 Handan Road, Shanghai 200433, P.R.China. Tel: ++86-2151370613; Fax:++86-21-513201101

**Keywords:** carboxylic acids, ethyl iodide, Ru_3_(CO)_12_, silanes, silylation

## Abstract

Ru_3_(CO)_12_/EtI has been found to be an efficient catalyst system for the dehydrosilylation of carboxylic acids with silanes. In the presence of 1 mol% Ru_3_(CO)_12_ and 4 mol% EtI, dehydrosilylation reactions in toluene afforded the corresponding silyl esters at 100 °C in good and high yields.

## Introduction

Polymers composed of nucleophilically-labile silyl ester bonds in the main chain are being studied as a new type of degradable functional polymers with the potential for an extremely broad range of degradation behavior through variation in the functionalities attached to the silicon atom. In the design of degradable materials, the physical and mechanical properties must be considered for performance in serving the expected function, while degradation rate and degradation products are also very important. Since the lability of a silyl ester linkage is dramatically affected by the substituents attached to the silicon atom, poly(silyl ester)s were found to be an ideal family of degradable polymers [1]. Also, multifunctional silyl esters have been found to be ideal cross-linking agents since they require only mild reaction conditions, especially for silicone elastomers. The demand for degradable poly(silyl ester)s has been increasing greatly due to biomedical field and environmental concerns [2–4]. Obviously, silyl esters are very important intermediates for the preparation of easily degradable functional poly(silyl ester)s, widely utilized as gene delivery carriers, matrices for drug delivery, biodegradable surgical devices, and recyclable materials [2–12]. To develop simple, economical and practical protocols for the conversion of carboxylic acids into silyl esters is not only required in normal organic synthesis procedure, but is also a prerequisite for the accurate performance of gas-chromatographic analyses in organic and biological chemistry [13–14]. From the viewpoint of synthetic chemistry, the ideal protecting group for an active-hydrogen moiety such as carboxylic acid should be attached in high yield, be stable towards severe reaction conditions and, at the same time, be selectively removable in the presence of other functional groups carrying different protecting groups. Indeed, silylation of carboxylic acids is a useful method for their protection because deprotection of silyl esters is easily achieved under mild reaction conditions [15–18].

Generally, silyl esters are made by the coupling of carboxylic acids and chlorosilanes [19–24]. Unavoidably, HCl is formed in these procedures, and a stoichiometric or even an excess amount of bases such as amines or ammonia is needed to consume the HCl gas formed. Since chlorosilanes themselves are produced by the chlorination of silanes, either with chlorine gas [7] or with hydrochloric acid under Pd/C catalysis [25–26], the synthesis of silyl esters from the corresponding silanes requires two reaction steps. Some newer synthetic protocols to silyl esters have been developed and a lot of literature focuses itself on the transition metal-catalyzed cross-coupling of an active hydrogen-moiety containing substances such as water and alcohols with silanes [27]. There are still few examples of dehydrogenative coupling reaction of carboxylic acids with silanes. Silylating agents such as allyltrimethylsilane, hexamethyldisilazane, aminosilanes, *N*-trimethylsilylalkanamines, *N*-trimethylsilyl-2-oxazolidinone, *N*-trimethylsilylacetamide, or trialkylsilyl 2-methallylprop-2-ene-1-sulfinates have been extensively utilized for the transformation of carboxylic acids into the desired silyl esters [28–43]. However, some shortcomings have been noted in these reported methods. The silylations of carboxylic acids with hexamethyldisilazane usually require prolonged reaction time under heating and continuous removal of ammonia or amine formed therein and the silylating agents are expensive. A few examples have been disclosed for dehydrosilylation reactions catalyzed by metal salts such as zinc chloride [26] or, more frequently, by transition metals and metal complexes such as [CuH(Ph_3_P)] [34], HPtCl_6_, Rh and Pd [36–44], Co_2_(CO)_6_ [45], Cu(Ph_3_P)_3_Cl [46] and promoted by organocatalyst such as triphenylphosphine [47]. Generally, catalysts such as transition metals are expensive. [CuH(Ph_3_P)] requires a multiple-step synthetic approach and *in-situ* generation protocols.

## Results and Discussion

**Scheme 1:**
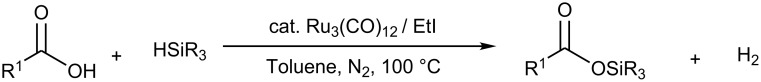
Dehydrogenative silyl ester synthesis with Ru_3_(CO)_12_/EtI.

In this communication, we wish to report the first finding that a catalytic system of dodecacarbonyltriruthenium and ethyl iodide [Ru_3_(CO)_12_/EtI] effectively promotes the dehydrogenative coupling of carboxylic acids with silanes, yielding the corresponding silyl esters selectively. The results are summarized in Scheme 1 and Table 1–Table 4.

**Table 1:** Ru_3_(CO)_12_-catalyzed dehydrocoupling of propionic acid with triethylsilane in toluene^a^.

Run	Ru_3_(CO)_12_ (mol%)	EtI (mol%)	Temp. (°C)	Time (h)	GC ratio (%)^b,c^	
	
					HSiEt_3_	CH_3_CH_2_CO_2_SiEt_3_

1	0.25	4	100	12	66	34
2	0.5	4	100	12	52	48
3	1	4	100	8	0	100 (95)
4	2	4	100	8	0	100 (93)
5	4	4	100	8	0	100 (94)
6	8	4	100	8	0	100 (92)
7	1	8	100	8	0	100 (92)
8	1	2	100	12	22	78
9	1	4	20	24	100	0
10	1	4	40	24	82	18
11	1	4	60	24	70	30
12	1	4	80	24	61	39

^a^Propionic acid (20 mmol), triethylsilane (20 mmol). ^b^GC ratio. ^c^Isolated yield in parentheses.

Dehydrogenative coupling reactions were carried out by heating a mixture of carboxylic acid, silane and a catalytic amount of Ru_3_(CO)_12_/EtI in solvents under a nitrogen atmosphere for several hours (Scheme 1, Table 1–Table 4, dehydrocoupling reaction was monitored by GC). The transformation of propionic acid with triethylsilane was employed as a model to optimize the reaction conditions.

The dehydrogenative coupling was found to be finished after 8 h at 100 °C, in the presence of 1 mol% Ru_3_(CO)_12_ and 4 mol% EtI in toluene, giving the corresponding triethylsilyl propionate in 95% yield (Table 1, Run 3). When the amount of Ru_3_(CO)_12_ was increased to 2, 4 or even 8 mol%, the product yields were 92–95% (Table 1, Runs 4–6). The reaction proceeded more slowly, however, when the amount of Ru_3_(CO)_12_ was decreased (0.25 or 0.5 mol%), where 66% and 52% of Et_3_SiH was found to be unreacted (GC ratio), even after 12 h at 100 °C (Table 1, Runs 1 and 2).

The reaction went more slowly when carried out at 20 °C, 40 °C, 60 °C or 80 °C. Thus, significant amount of Et_3_SiH was found to be unreacted (82% at 40 °C, 70% at 60 °C, 61% at 80 °C) even being heated for 24 h (Table 1, Runs 10–12). No silyl ester was detected at 20 °C even after 24 h and all of the Et_3_SiH was recovered (Table 1, Run 9). When the amount of EtI was increased to 8 mol%, the product yields were 94% (Table 1, Run 7). The reaction went more slowly when the amount of EtI was decreased to 2 mol%, where 22% of Et_3_SiH remained unreacted, even after 12 h at 100 °C (Table 1, Run 8).

Dehydrosilylation in different solvents was also investigated (Table 2). In xylene, ethylbenzene, *tert*-butylbenzene, mesitylene, *n*-octane, diethylene glycol diethyl ether, and anisole, the dehydrogenative coupling is slightly slower compared with toluene, and some amount of Et_3_SiH was detected (12–22%) (Table 2, Runs 2–8). In *N,N*-dimethylformamide (DMF) and *N,N*-dimethylacetamide (DMAc), the dehydrocoupling failed to reach completion being heated at 100 °C for 48 h, and significant amounts of Et_3_SiH were detected (Table 2, Runs 9 and 10).

**Table 2:** Ru_3_(CO)_12_-catalyzed dehydrocoupling of propionic acid with triethylsilane in different solvents.

Run	Solvent	Time (h)	GC ratio (%)	
	
			HSiEt_3_	CH_3_CH_2_CO_2_SiEt_3_

1	Toluene	8	0	100
2	*n*-Octane	24	22	78
3	Xylene	24	17	83
4	Ethylbenzene	24	15	85
5	*tert*-Butylbenzene	24	14	86
6	Mesitylene	24	12	88
7	Anisole	24	14	86
8	Diethylene glycol diethyl ether	24	20	80
9	DMF	24	67	33
10	DMAc	24	61	39

Other Ru complexes were also tested as catalysts for the dehydrogenative coupling and the results were summarized in Table 3 (usually at 1 mol% of Ru complex, 4 mol% EtI, toluene, 100 °C). In the case of using [RuCl_2_(CO)_3_]_2_, RuCl_2_(CO)_2_(PPh_3_)_2_, Ru(acac)_3_, RuCl_2_(2,2′-bipy)_3_ and RuCl_2_(PPh_3_)_3_ as catalysts, 9–27% of Et_3_SiH was still detected after heating for 24 h at 100 °C (Table 3, Runs 4–8). In the presence of 4 mol% of ethyl bromide, the dehydrogenation was slightly slower compared with EtI, and a small amount of Et_3_SiH was still found. Without EtI, the dehydrocoupling was sluggish and 81% of Et_3_SiH were detected even being heated for 24 h at 100 °C (Table 3, Run 3).

**Table 3:** Catalyst-screening for the dehydrocoupling of propionic acid with triethylsilane in toluene at 100 °C.

Run	Catalyst	Time (h)	GC ratio (%)	
	
			HSiEt_3_	CH_3_CH_2_CO_2_SiEt_3_

1	Ru_3_(CO)_12_	8	0	100^a^
2	Ru_3_(CO)_12_	8	9	91^b^
3	Ru_3_(CO)_12_	24	81	19^c^
4	[RuCl_2_(CO)_3_]_2_	24	9	11^a^
5	RuCl_2_(CO)_2 _(PPh_3_)_2_	24	11	86^a^
6	RuCl_2_(PPh_3_)_3_	24	10	84^a^
7	Ru(acac)_3_	24	27	73^a^
8	RuCl_2_(2,2′-bipy)_3_ 6H_2_O	24	25	75^a^

^a^Additive: 4 mol% EtI, ^b^Additive: 4 mol% EtBr, ^c^no Additive.

Treatment of a number of carboxylic acids and silanes such as triethylsilane, tri-*n*-propylsilane (*n*-Pr_3_SiH), tri-iso-propylsilane (iso-Pr_3_SiH), tri-*n*-butylsilane (*n*-Bu_3_SiH) or *tert*-butyldimethylsilane (*tert*-BuMe_2_SiH) afforded the corresponding silyl esters in good and excellent yields (all with 1 mol% Ru_3_(CO)_12_ and 4 mol% EtI in toluene at 100 °C, Table 4). In the case of nitro-, bromo- and chlorobenzoic acid, the expected silyl esters were obtained in 85–95% yields, free of dehalogenated or over-reduced by-products (Table 4, Runs 12–16 and 23).

**Table 4:** Ru_3_(CO)_12_-catalyzed dehydrocoupling of carboxylic acids with silanes^a^.

Run	Acid	Silane	Time (h)	Product	Yield (%)^b^

1	CH_3_CO_2_H	Et_3_SiH	8	CH_3_CO_2_SiEt_3_	94 [30]
2	CH_3_CO_2_H	(*n*-Pr)_3_SiH	8	CH_3_CO_2_SiPr*^n^*_3_	92 [48]
3	CH_3_CO_2_H	(*n*-Bu)_3_SiH	8	CH_3_CO_2_SiBu*^n^*_3_	93 [48]
4	CH_3_CH_2_CO_2_H	Et_3_SiH	8	CH_3_CH_2_CO_2_SiEt_3_	95 [28]
5	CH_3_CH_2_CO_2_H	(*n*-Pr)_3_SiH	8	CH_3_CH_2_CO_2_SiPr*^n^*_3_	89 [48]
6	CH_3_CH_2_CO_2_H	(*n*-Bu)_3_SiH	8	CH_3_CH_2_CO_2_SiBu*^n^*_3_	91 [49]
7	CH_3_(CH_2_)_8_CO_2_H	(iso-Pr)_3_SiH	9	CH_3_(CH_2_)_8_CO_2_SiPr^i^_3_	90 [18]
8	C_6_H_5_CH_2_CO_2_H	Et_3_SiH	8	C_6_H_5_CH_2_CO_2_SiEt_3_	92 [28]
9	C_6_H_5_CH_2_CO_2_H	(iso-Pr)_3_SiH	9	C_6_H_5_CH_2_CO_2_SiPr^i^_3_	93 [18]
10	C_6_H_5_CH_2_CO_2_H	*tert*-BuMe_2_SiH	10	C_6_H_5_CH_2_CO_2_SiMe_2_Bu*^t^*	85 [18]
11	C_6_H_5_CH(Me)CO_2_H	(iso-Pr)_3_SiH	9	C_6_H_5_CH(Me)CO_2_SiPr^i^_3_	92 [18]
12	3-BrC_6_H_4_CO_2_H	(iso-Pr)_3_SiH	8	3-BrC_6_H_4_CO_2_SiPr^i^_3_	92 [50]
13	3-BrC_6_H_4_CO_2_H	*tert*-BuMe_2_SiH	10	3-BrC_6_H_4_CO_2_SiMe_2_Bu*^t^*	86 [50]
14	3-ClC_6_H_4_CO_2_H	(iso-Pr)_3_SiH	8	3-ClC_6_H_4_CO_2_SiPr^i^_3_	91 [50]
15	3-ClC_6_H_4_CO_2_H	*tert*-BuMe_2_SiH	10	3-ClC_6_H_4_CO_2_SiMe_2_Bu*^t^*	85 [50]
16	4-ClC_6_H_4_CO_2_H	Et_3_SiH	8	4-ClC_6_H_4_CO_2_SiEt_3_	91 [51]
17	C_6_H_5_CO_2_H	Et_3_SiH	8	C_6_H_5_CO_2_SiEt_3_	93 [52]
18	C_6_H_5_CO_2_H	(*n*-Pr)_3_SiH	8	C_6_H_5_CO_2_SiPr*^n^*_3_	92 [53]
19	C_6_H_5_CO_2_H	(iso-Pr)_3_SiH	9	C_6_H_5_CO_2_SiPr^i^_3_	95 [18]
20	C_6_H_5_CO_2_H	(*n*-Bu)_3_SiH	8	C_6_H_5_CO_2_SiBu*^n^*_3_	91 [54]
21	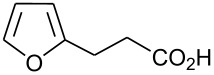	Et_3_SiH	8	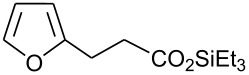	94 [55]
22	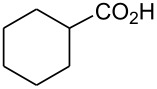	(iso-Pr)_3_SiH	9	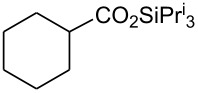	93 [18]
23	4-O_2_NC_6_H_4_CO_2_H	*tert*-BuMe_2_SiH	10	4-O_2_NC_6_H_4_CO_2_SiMe_2_Bu*^t^*	88 [56]

^a^Carboxylic acid (20 mmol), silane (20 mmol), Ru_3_(CO)_12_ (0.2 mmol, 1 mol%), EtI (4 mol%), 100 °C. ^b^Isolated yield.

## Conclusion

In conclusion, we have demonstrated that Ru_3_(CO)_12_/EtI is an efficient catalytic system for the dehydrogenative cross-coupling of carboxylic acids with silanes. The dehydrogenative cross-coupling reactions proceed efficiently to give the corresponding silyl esters in good and excellent yields. No over-reduced silyl esters are formed in the case of coupling nitro-, bromo-, and chlorobenzoic acid with silanes. We believe that the Ru_3_(CO)_12_/EtI-catalyzed dehydrosilylation of carboxylic acids with silanes provides another important protocol for a one-step, highly selective, atom-economical and efficient synthetic method. We are currently broadening the scope of this dehydrosilylation of carboxylic acids and silanes in our laboratory and the results will be published elsewhere.

## Experimental

To a mixture of propionic acid (40 mmol, 2.96 g), and triethylsilane (40 mmol, 4.64 g) in toluene (20 ml) was added Ru_3_(CO)_12_ (0.4 mmol, 0.01 equiv) and EtI (2.0 mmol, 0.05 equiv) at room temperature under a nitrogen atmosphere. The reaction mixture was stirred at 100 °C for 8 hours (monitored by GC). The desired triethylsilyl propionate was obtained as a colourless oil (yield: 95%) after distillation under reduced pressure (Table 1, Run 3). **Triethylsilyl propionate** [29]: IR (neat): 686, 742, 826, 995, 1063, 1240, 1410, 1466, 1718, 2872, 2952 cm^−1^. ^1^H NMR (400 MHz, CDCl_3_): δ 0.74 (6H, q, ^3^*J* 7.8 Hz), 0.95 (9H, t, ^3^*J* 7.8 Hz), 1.14 (3H, t, ^3^*J* 7.6 Hz), 2.36 (2H, q, ^3^*J* 7.6 Hz). ^13^C NMR (100 MHz, CDCl_3_): 4.46, 6.48, 9.32, 28.44, 175.26.

All of the silyl esters are known compounds and were compared with authentic samples [prepared by cross-coupling of carboxylic acids and chlorosilanes in the presence of a base such as triethylamine or imidazole (*tert*-butylsilyl esters) in dichloromethane] and were identified on the basis of their IR, ^1^H NMR, ^13^C NMR and GC-MS spectral data.
